# Knowledge Mapping of Research on Land Use Change and Food Security: A Visual Analysis Using CiteSpace and VOSviewer

**DOI:** 10.3390/ijerph182413065

**Published:** 2021-12-10

**Authors:** Peng Cheng, Houtian Tang, Yue Dong, Ke Liu, Ping Jiang, Yaolin Liu

**Affiliations:** 1 College of Resource and Environmental Sciences, Wuhan University, Wuhan 430079, China; chengpeng1141@whu.edu.cn (P.C.); dongyue@whu.edu.cn (Y.D.); 00007101@whu.edu.cn (Y.L.); 2 School of Public Administration, Central South University, Changsha 410083, China; tanghoutian@csu.edu.cn; 3 Graduate School of Huazhong Agricultural University, Wuhan 430070, China; liuke_1996@163.com; 4 Key Laboratory of Geographic Information System, Ministry of Education, Wuhan University, Wuhan 430079, China

**Keywords:** land use change, food security, visual analysis, CtieSpace, VOSviewer, progress and frontier

## Abstract

Many scholars have conducted in-depth research on the theme of land use change and food security, and formed fruitful research results, but there is a lack of quantitative analysis and comprehensive evaluation of research achievements. Therefore, based on the relevant literature on the theme of land use change and food security in the core collection of the Web of Science (WOS) database, this paper takes the advantage of CiteSpace and VOSviewer bibliometric software to draw the cooperative network and keyword cooccurrence map to analyze the research progress and frontier. The results reveal that: (1) The research started in 1999 and can be divided into three stages: initial research, rapid development, and a stable in-depth stage. This topic has increasingly become a research hotspot in the academic community. (2) The distribution of research institutions is concentrated and forms a small cluster, and the research networks between developed and developing countries have been established, and developed countries are in the core position, but the cooperation network is not prominent. (3) The research content is becoming increasingly organized and systematic, and the research hot topics are divided into seven aspects. (4) The research area of the subject covers multiple levels, such as global, national, and specific natural geographical regions, and has formed a research system of geographic information technology and satellite remote sensing technology. It also presents the trend of cross integration with economics, land management and soil science. In the future, theoretical innovation still needs to be strengthened, and we should strengthen the research on the impact of agricultural chemical fertilizers on food security and study the impact of urban expansion on land use change.

## 1. Introduction

Land use change and its impact on food security have become one of the frontiers and hot topics studied by scholars worldwide [1,2,3,4]. With the development of human society, the structure, depth and intensity of land use are constantly changing, which not only affects biodiversity but also has a great impact on human food security [5]. The issue of “food security” was first put forward by the United Nations International Food and Agriculture Organization at the First World Food Summit in November 1974. The definition of food security is to ensure that anyone can obtain enough food for survival and health at any time, including food supply, food access, food stability and food utilization [6,7]. Over the years, food security has been a major issue related to the overall political and economic situation of a country or region. In particular, regional food production and food security have become hot issues of concern to governments and scholars [8,9,10]. According to the prediction of the United Nations [11], the global population will exceed 9.8 billion in 2050, food demand will increase by more than 50%, and food problems will be extremely serious [12]. However, there are many factors affecting grain production, including institutional and policy innovation, scientific and technological progress, material and labor investment, climate change, but cultivated land resources in land resources are the most important factor in grain production [13,14]. Cultivated land resources are the most basic natural condition of agricultural production. Food security is closely related to changes in cultivated land. The change in the quantity and quality of cultivated land directly affects grain output and then affects the effective supply of grain and the level of food security [15,16].

In recent years, research related to the theme of land use change and food security has emerged in the academic community, mainly combined with issues of climate change, carbon emissions, agricultural intensification [5,17,18]. For example, Galeana-Pizaña et al. [1] used a GIS-based food environmental efficiency (FEE) index to evaluate the trend of land use change and regional food security, and the FEE index proved useful assessment of land use policies. Moore et al. [19] used the regional climate model to compare the impacts of projected future greenhouse gases and future land use change on spatial variability of grain yields in East Africa. These show that this theme is an evolving knowledge field, but there is a lack of systematic review of research results. Accurately understanding the research progress and academic trends of land use change and food security is of great significance for carrying out follow-up research. Therefore, based on the core collection of the Web of Science (WOS) database, this paper comprehensively uses the advantages of CiteSpace and VOSviewer software to conduct bibliometric analysis, systematically and visually analyze and summarize the literature in the fields of land use change and food security, and explore the status of research. This research field objectively reveals the trends, accurately evaluates the research progress on land use change and food security and provides a reference for combining the research framework and expanding new ideas and methods in this field.

## 2. Data Collection and Research Methods

### 2.1. Data Collection

The data on the relevant literature used in this paper come from the core collection of the WOS database (http://apps.webofknowledge.com, accessed on 6 September 2021) and adopt the method of group retrieval. The WOS Citation database is an information retrieval platform developed by Thomson Reuters of the United States. With the Science Citation Index, Social Science Citation Index, and Arts and Humanities Citation Index as the core, it contains more than 9000 world authoritative and influential academic journals, and the documents in the database have high authority in the academic community [20,21]. The search prerequisites of this research are set as follows: (TS = “land use change” and “grain security”) OR (TS = “land use change” and “food security”), TS is the theme, time spans are unlimited, the language is “English”, and the literature types are “article” and “review”. There were 628 literature records related to the subject that were retrieved. To avoid duplicate literature, CiteSpace’s deduplication function was used for inspection, and no duplicate publications were found.

### 2.2. Research Methods

As an auxiliary procedure of bibliometrics, science mapping provides a spatial representation of network structures. Science mapping involves the interdisciplinary fields of applied mathematics, information science and computer science. It is a new development of scientometrics and information metrology. In recent years, with the rapid development of computer science, many scholars have used various science mapping tools to analyze the potential dynamic mechanism of discipline evolution [22,23,24]. CiteSpace and VOSviewer software are two powerful and complementary science mapping analysis tools. CiteSpace (https://sourceforge.net/projects/citespace, accessed on 6 September 2021) is a Java-based application software proposed by Professor Chen of Drexel University in 2004. It is based on the co-citation analysis theory and pathfinder, minimum spanning trees algorithm to make a quantitative analysis of the literature in specific fields, which is used to analyze and visualize the emerging trends and patterns in the knowledge field of scientific publications [25,26]. It has unique advantages in literature keyword analysis, cluster analysis, subject words, author information. VOSviewer (https://www.vosviewer.com, accessed on 6 September 2021) is also a literature analysis and knowledge visualization software tool developed by van Eck and Waltman of the Centre for Science and Technology Studies at Leiden University [27]. It can realize the construction and visualization of the keyword cooccurrence network in various fields. Compared with other visualization software, VOSviewer software has advantages in processing big data and drawing images, which can more clearly show the hot spots and topics in the research field [28].

## 3. Results

### 3.1. Trend Analysis of Literature Publication

**Figure 1:**
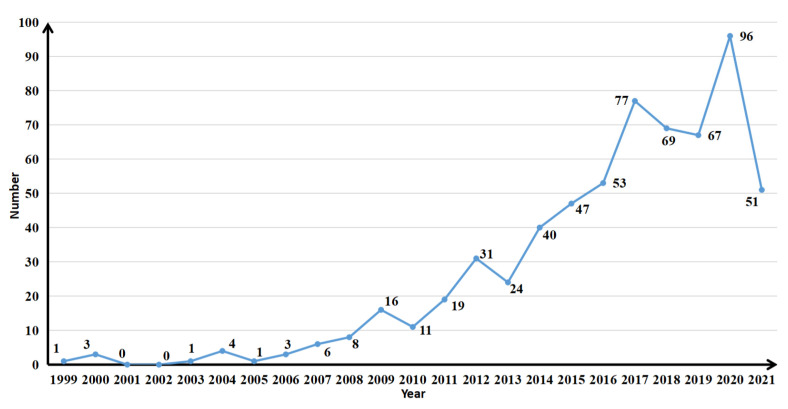
Number of articles published annually on the theme of land use change and food security.

The annual distribution of the number of published articles can reflect the research level and degree of development of a certain discipline [29]. The number of published articles on land use change and food security is shown in Figure 1. From 1999 to 6 September 2021, the number of published studies on land use change and food security showed a stable growth trend on the whole, which experienced three stages, i.e., initial research, rapid development, and a stable in-depth stage.

The initial stage of the research (1999–2008): At this stage, the number of relevant research studies was relatively small, the research topic was relatively limited, and the number of research scholars in this field was also small, mainly because there was not much research on land use change and food security. Before this stage, scholars studied the theme of land use change or food security separately. In 1999, Sporton et al. first studied the theme of land use change and food security [30]. Subsequently, Murdiyarso and Verburg et al. paid more attention to this research topic [31,32].

The rapid development stage of the research (2009–2017): During this stage, scholars showed great interest in the research topic, the number of published articles continued to increase, the research questions and perspectives were further expanded, and several leading studies emerged, such as Alexander and Verburg et al. [33,34]. In 2009, famous scholars such as Khan, Garnett, Mertz and Yan et al. [35,36,37,38] published a series of high-level papers, meanwhile, the number of people suffering from hunger in the world will reach 1.02 billion in 2009 announced by the United Nations, reversing the continuous decline of hungry people, which made the research on land use change and food security widely concerned in the academic community. With the intensification of global land use change and food security, scholars researched climate change, biodiversity, policy development, agriculture, greenhouse gas emissions, and the research contents and methods were further enriched [19,39].

Stable in-depth stage of the research (2018-present): During this stage, the literature publication trend was relatively smooth, and the research content and perspective gradually increased in depth. The research perspective has focused on both macro and micro issues, including population growth, land systems, rural development, soil organic carbon, and life cycle assessment, in the research agenda of land use change and food security, and the overall research has continued to deepen [40,41].

### 3.2. Network Analysis of Author Cooperation, Institutional Cooperation and National Cooperation

#### 3.2.1. Analysis of Author Cooperation Network

**Figure 2:**
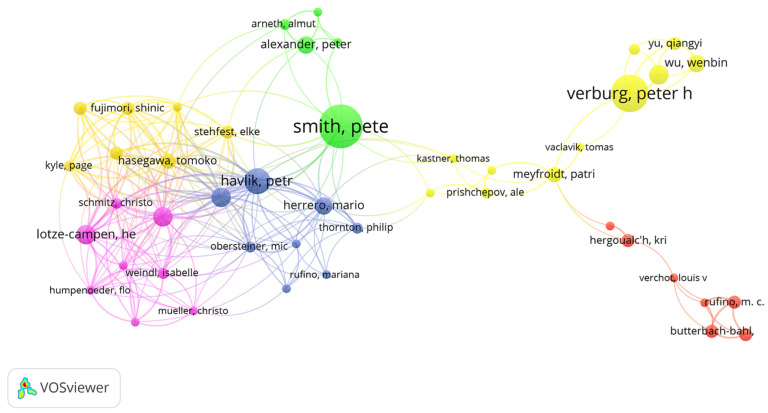
Author cooperation network map.

By analyzing the author’s cooperation network [42], we can determine the strength of representative scholars and core research teams in the field of land use change and food security. VOSviewer software was used to overlay and visualize the author collaborative networks with more than 5 published articles. Through the color gradient, it can intuitively reflect the cooperation of various scholars in recent years (Figure 2) and present the author information with the number of published articles for the top 20 publications (Table 1). We found that the authors with a large number of published articles showed obvious network characteristics, mainly including the cooperative network of Verburg, Smith, Havlik and Popp. This indicates that these are core authors who have developed a high-yield author research team in the field of land use change and food security that has initially formed a scale. According to the Price Law [43], the formula for calculating the minimum number of published articles of core authors in a field is $m=0.749\times \sqrt{n_{\max}}=$ 2.996 (where $n_{\max}$ is the number of published articles of the top 1 author). Therefore, authors with more than three published articles are regarded as the core authors in this field. The top three scholars in the number of published articles are Verburg (16 articles), Smith (13 articles) and Havlik (10 articles). According to the data, there are 43 core authors and 206 articles, accounting for 32.8% of the total articles published in this field, which is less than the standard of 50% of the Price Law. This shows that after more than 20 years of development, the core author group in the field of land use change and food security has initially formed, but still needs further development.

#### 3.2.2. Analysis of Institutional Cooperation Network

**Figure 3:**
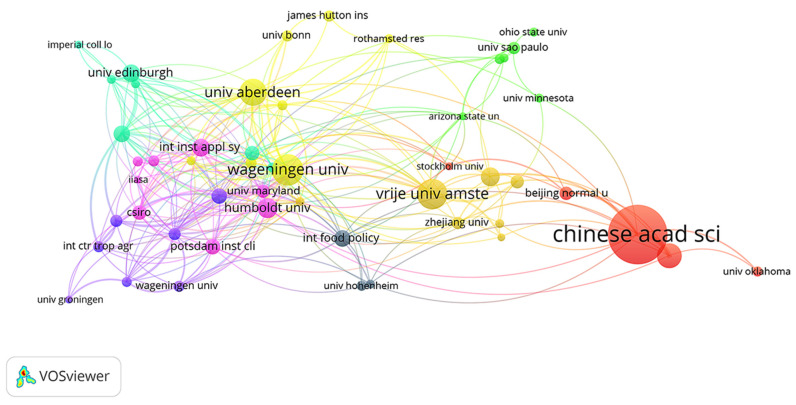
Institutional cooperation network map.

Taking the research institution as the node for visual analysis, we can obtain the cooperation network map of the research institution (Figure 3) and show the network with connections. According to the information of the top 20 major research institutions (Table 2), the Chinese Academy of Sciences has the highest number of published articles (52), followed by Vrije University Amsterdam (26) and Wageningen University (22), and a research network has been formed of these three research institutions as the core. This shows that these institutions have strong scientific research and influence in the field, and there are cooperative relations and large-scale collaborations between the different institutions. It is worth noting that the reason for the highest number of documents issued by the Chinese Academy of Sciences may be related to China’s national conditions. The main reasons include the following points: (1) In terms of policy, the Chinese Government has put forward the “red line of 1.8 billion mu of cultivated land” and other cultivated land protection policies to control land use changes and ensure food security. (2) In terms of economics, the Chinese Government has adjusted agricultural protection policies, increased investment in agricultural science and technology, and continuously improved the rate of grain self-sufficiency. (3) In terms of society, China is a populous country in the world, it is required to ensure food supply and firmly put its rice bowl in its own hands. (4) In terms of the environment, the deterioration of land and other production factors had a great impact on food security. To ensure food security, the Government has always taken measures to prevent land resource degradation and improve the ecological environment.

In Figure 3, the research institutions are in a local aggregation state, indicating that the distribution of research institutions is relatively concentrated and that a small aggregation cluster is formed; that is, there are some cooperative relations among institutions. Generally, there are a large number of research institutions related to the theme of land use change and food security, but a large cross-national institutional cooperation group has not yet formed.

#### 3.2.3. Analysis of Country Cooperation Network

**Figure 4:**
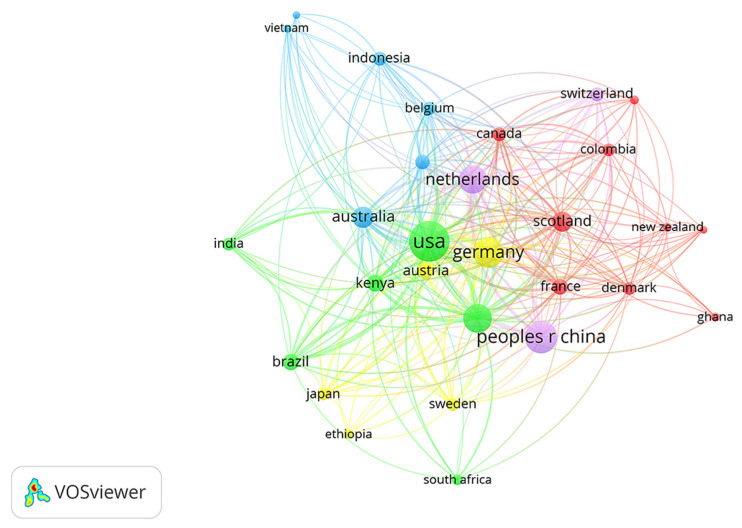
Country cooperation network map.

According to Figure 4 and Table 3, there are more than 100 articles published in the USA, China, and Germany, which is significantly higher than that in other countries. The number of articles published in these three countries accounted for 31.84%, 19.9% and 18.15% of the total number of articles published in this field, respectively. It can be seen from the connectivity in Figure 4 that the connections between nodes are dense and complex, indicating that there are many cooperative relations between different countries. In Figure 4, purple appears at the edge of some nodes, indicating that the centrality is ≥0.1, which also indicates that the node is in an important position within the network structure. Among them, the centrality values of the USA (0.58), Germany (0.13), England (0.11) and France (0.11) are higher than 0.1, indicating that these countries are in a relatively core area in the research field of land use change and food security and that the relevant research studies have a significant impact on this field.

### 3.3. Analysis of Hot Research Topics and Frontiers Trending

#### 3.3.1. Analysis of Hot Research Topics

**Figure 5:**
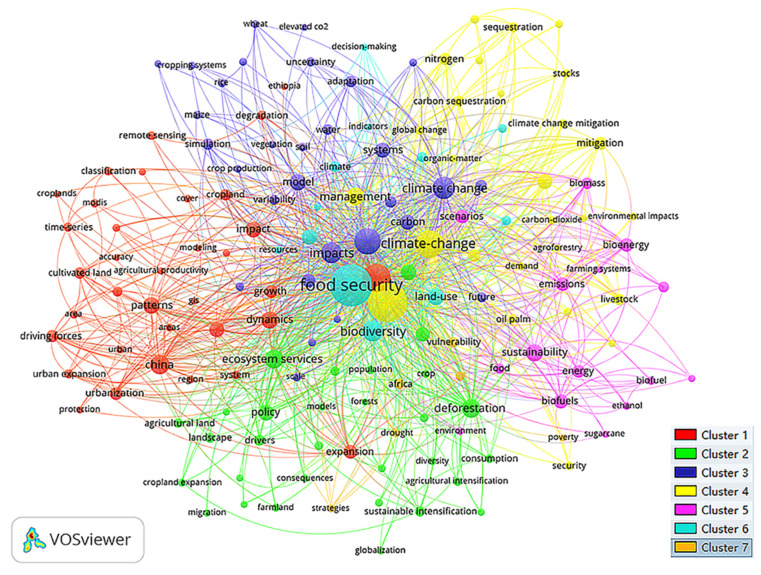
Keyword cooccurrence network map.

Keywords capture the core idea of the article. Through the research on keywords in a field, we can quickly grasp the hot topics in the field [44]. In this study, VOSviewer software was used to visualize keywords. Nodes in the knowledge map represent keywords. The larger the node is, the higher the frequency, and the lines between nodes represent the cooccurrence of particular keywords. In addition, in the VOSviewer knowledge map, different colors represent different clusters, and the same color represents the same cluster. By analyzing the keyword cooccurrence knowledge map (Figure 5), we find that the whole keyword knowledge map takes “food security”, “land use change” and “climate change” as the core, producing a radial shape. Considering that high-frequency keywords can be clearly displayed, a total of 279 high-frequency keywords are obtained with the threshold of five of each keyword. The cooccurrence map of keywords is relatively clear, and the top 20 high-frequency keywords are shown in Table 4. As seen from Figure 5 and Table 4, “land use change” (185), “food security” (141), “climate change” (119), “impact” (102) and other high-frequency keywords constitute representative terms in this field. In terms of layout, these high-frequency keywords are also key hub nodes. Other nodes around them have together formed the hot cutting-edge research topics in this field in recent years.

**Figure 6:**
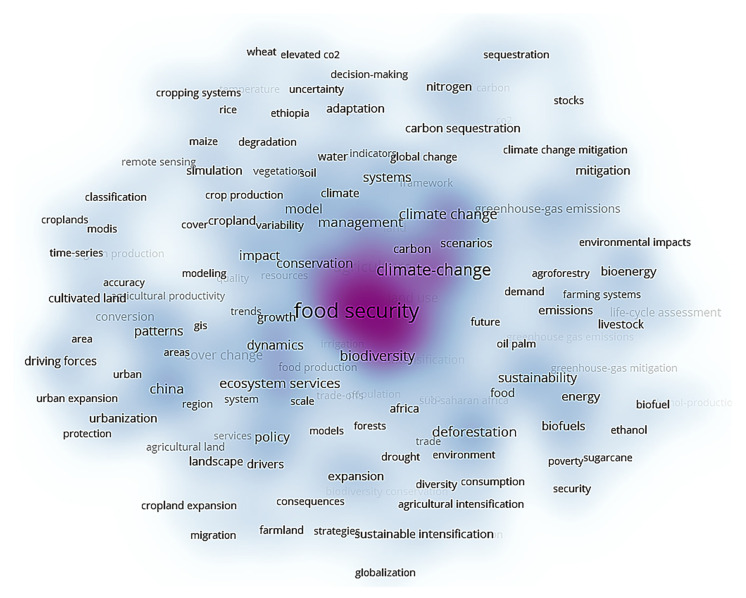
Keyword cooccurrence clustering density map produced by VOSviewer software.

To refine the research topics more intuitively and effectively in this field, we use the unique clustering density map function of VOSviewer software to visualize the keyword cooccurrence clustering results (Figure 6). In the cluster density map, the density of an element depends on the number and weight of its surrounding elements. From the cold tone to the warm tone, the representative clustering density gradually increases; that is, the frequency of keyword cooccurrence increases, and the heat of related research topics increases [45]. According to the clustering results in Figure 6, combined with professional knowledge, we can extract seven frontier hot topics in the current research field of land use change and food security (Table 5) and further analyze and discuss the research contents and important achievements of each frontier hot topic.

##### Climate Change and Carbon Emissions

With excessive carbon emissions produced in the process of human production and consumption, global warming and abnormal climate events frequently occur, which have a direct impact on land use, especially on changes in cultivated land area, threatening global food security [46,47,48]. At present, with the continuous intensification of abnormal climate change, the geographical distribution of food-deficient areas will further expand. At the same time, grain production areas have been chronically affected by energy crops, feed crops, forestry and other economic crops, as well as the continuous expansion of vegetation areas caused by climate warming, which forces people to reduce the land allocated to grain production, thus, causing a global food supply crisis [49,50,51]. Hasegawa et al. built a comprehensive assessment model of the impact of climate change mitigation policies on food security [52]. The research found that if mitigation policies to address climate change are strictly implemented, they will have a huge negative impact on global food production and consumption, especially in low-income countries in Africa and South Asia. Moreover, Nobre and Beltrán-Tolosa et al. found that the development of traditional agriculture and animal husbandry will inevitably reduce the area of vegetation coverage, resulting in environmental problems such as soil and water loss and soil erosion, leading to drought with climate change, thus affecting the production of the main food crops [53,54,55]. Relevant studies show that by the end of this century, due to the impact of climate change, grain prices may rise by 110% or more over the prices in the baseline year. Similarly, Hasegawa and Popp et al. also confirmed that food prices in parts of Asia and Africa will be more affected, increasing the potential risk of a food crisis [49,56]. Therefore, the impact of climate change and carbon emissions on land use change and food security will still be one of the key topics that scholars continue to pay attention to in the future [57,58].

##### Sustainable Land Management Policy

A sustainable land management policy can help mitigate climate change, protect the land from soil erosion and ensure food security. Its core is to emphasize the resilience of management methods, that is, to seek maximum synergy through the combination of different land management policies [59,60]. Russo and Pavone believe that the mitigation potential of multiple land management policies that work together on the same land is generally greater than that of a single policy [61]. Moreover, Dax et al. also confirm that the combination of multiple land management policies can save resources, enhance social resilience and promote ecological restoration to better mitigate and adapt to climate change, prevent desertification and land degradation, and strengthen food security [62]. For example, (1) strengthening the combination of fire management and afforestation can increase land carbon sequestration, enhance the potential to mitigate climate change and land degradation, reduce management costs and ensure food production areas [63]. (2) Reducing food waste and a carnivorous diet will help to reduce carbon emissions, achieve sustainable land use management, and ensure food security and low carbon emissions [64]. (3) The construction of urban green infrastructure is also a solution to mitigate climate change. Through measures such as vertical greening, roof gardens, suburban agriculture and vertical agriculture, it can not only meet some food needs of urban residents but may also reduce the pressure of rural land food production and land degradation [65,66]. (4) In addition, improving land market management policies, ensuring land ownership and integrating environmental costs into food security and ecological compensation will help to achieve sustainable land management and eliminate poverty to achieve food security with stable food production [67,68]. A successful sustainable land management policy requires the participation of more stakeholders, especially local stakeholders such as local farmers and community residents who are easy to ignore, which can fully mobilize their enthusiasm to understand and practice land management policies [69,70]. However, there are great differences in the actual situation in diverse regions. Therefore, in future research, scholars should realistically build sustainable land management policies suitable for each region to achieve the stable production of food crops and ensure food security.

##### Agricultural Intensive Development

In the face of the food security crisis, although the development of marginal ecological land can improve food output in the short term, this process is mostly irreversible, and the opportunity for agricultural land expansion is limited [71]. Excessive exploitation of natural resources can easily lead to land degradation and reduction of ecological land area, resulting in greater social and ecological costs [12]. Therefore, the intensive development of land use is considered to be the fundamental approach to not only ensure the needs of human land products and functions but also to effectively reduce marginal land development and protect the ecological environment [72,73]. Compared with the traditional intensification realized by changing management practices and decisions, sustainable land use intensification has been widely discussed and explored as a necessary way to improve global food security and reduce ecological vulnerability and environmental pollution. Moreover, Charles and Struik et al. sustainable intensification can balance the competing demands for land use, improve ecosystem services and maintain biodiversity while increasing production to achieve common growth [74,75].

Research on agricultural intensification can be traced back to 1990. Vlek took sub-Saharan Africa as an example to explore the role of alternative soil fertility and other measures in agricultural production [76]. There are relatively many studies on sustainable intensification, focusing on the sustainable intensification of agricultural land, farms and agricultural production; the specific research contents include the conceptual connotation, empirical evaluation, impact mechanism, biodiversity, and improvement of soil organic matter [72,77]. For example, Wezel et al. [78] distinguished between the concepts of “ecological intensification”, “sustainable intensification” and “agricultural ecological intensification” and analyzed the subtle differences of the three concepts. Mulwa et al. [79] used the dynamic random effect probit model and the control function method to evaluate the vitality of adopting sustainable agricultural inputs and the effect of large grain traders strengthening the adoption of these sustainable agricultural inputs at the farm level. However, with the in-depth development of agricultural intensification, ecological and environmental problems have gradually appeared. How to stabilize food production under the condition of coordinating land use types and protecting the ecological environment still needs further research.

##### Land Degradation

Climate change has changed the process of surface change and terrestrial ecosystems and their composition, structure and function [80], triggered changes in land use, accelerated the process of desertification and land degradation in many areas, reduced agricultural output and agricultural income, and deeply affected the security of world food production. According to the relevant data released by the Intergovernmental Science-Policy Platform on Biodiversity and Ecosystem Services (IPBES) of the United Nations, human intervention has degraded the ecological function of approximately 80% of the world’s agricultural land, 10–20% of pasture land and 87% of wetlands, which brings economic losses ranging from 450 billion to 10.6 trillion US dollars to the global ecological service system every year; it also directly or indirectly affects the well-being of approximately 3.2 billion people around the world [81,82].

Land degradation seriously affects food production and distribution through soil erosion, the decline of land fertility and salinization [83]. Paoloni and Onorati found that it also directly threatens the well-being of the rural population, children and women and affects food security worldwide [84]. At present, a large number of studies have analyzed the factors of land degradation by exploring the driving force of land use change to analyze its impact on food security, especially from the aspects of geographical conditions, population characteristics, economic growth, road traffic, meteorological factors, Government policies, and technological evolution [75,85,86,87]. Among them, Prokop [86] analyzed the degree and type of land degradation of the Meghalaya Plateau through remote sensing data and found that the impact of different land degradation types and degrees on grain yield showed differentiated trends. In the face of land degradation, in response to the increase in food demand, Ranasinghe and Piyadasa [87] argue that we should integrate the main environmental, natural and socioeconomic factors in a region to build a productive land management system and explore an optimal mode of land production use to ensure the stable production of food. Land degradation is closely related to food production. Adopting sustainable land management policies not only effectively curbs the trend of land degradation and optimizes land use structure but also gives full play to the overall efficiency of different land types.

##### Renewable Bioenergy

With increasing attention given to energy security and ecological security, governments worldwide are pursuing multiple goals of energy security, reducing greenhouse gas emissions, and developing rural economies. Tian and Renzaho et al. found that the government have invested much money or established tax incentive mechanisms to develop renewable bioenergy represented by fuel ethanol and biodiesel to replace nonrenewable fossil fuels (coal, oil and natural gas) [88,89]. While the world vigorously advocates for the development of bioenergy to ensure energy security, the demand for land for bioenergy production is also increasing [90]. With the sharp rise of global food prices, whether bioenergy threatens food security is not only the focus of major international organizations and governments but also the main topic of debate within the academic community [46,91]. Although the use of bioenergy instead of fossil fuels can effectively reduce greenhouse gas emissions to a certain extent, the large-scale increase in bioenergy demand may also cause forest degradation and reduce food production [56,92]. Moreover, a large number of agricultural products are used to produce bioenergy, which greatly reduces the food supply in the international market and will inevitably lead to an increase in food prices [93], threatening global food security, especially the basic living needs of people in low-income countries with food shortages [94,95]. However, at present, there is no systematic research on how much-cultivated land is occupied by the development of bioenergy, what impact it has on land use change, how energy crops compete with other crop types at the household scale, and how to stabilize food production, which are worthy of further exploration by scholars in the future.

##### Food Production

Food security is a multidimensional security goal and is affected by many factors, among which food production is the most critical link in the food security system [96,97,98]. Land use change affects regional food production through changes in area and spatial location among different land use types, and temporal and spatial changes in cultivated land are one of the main forms of land use change [99], which affects the global food security supply [100]. At present, the research focus of most scholars is on quantifying the impact of cultivated land change on food security. However, due to the differences in research methods, regions and periods, the research results are also quite different [100]. For example, Wang et al. constructed the evaluation framework of “land food water” to quantify the impact of temporal and spatial changes in cultivated land on food production and water resource consumption and proposed the sustainable development policy of cultivated land and the optimal management policy of water resources [101].

In addition, the research results of some scholars show that grain production is affected by a variety of natural and socioeconomic factors, among which regional factors, family size, farming system, land use intensity, land tenure, climate change and environmental cost have a great influence on grain productivity. The actual grain yield is affected by the quantity and quality of cultivated land, climate, agricultural technology, and planting methods [13,102,103]. However, on the whole, although the current research helps to alleviate the contradiction between cultivated land change and food production, there are still some aspects to be optimized. For example, (1) the relevant research in this field is carried out at the national or a natural area level, which makes it difficult to guide practical work at the provincial level, and (2) weak supervision of newly reclaimed cultivated land and insufficient reserve resources of cultivated land easily leads to potential questions of food security production.

##### Agricultural Benefits

Global land use change is affected not only by climate change, land degradation and other factors but also by economic factors such as agricultural benefits [104]. The former is an irresistible natural factor, while the latter is the spontaneous change of land use types by farmers in pursuit of better comprehensive benefits [105]. In the environment of the market economy, farmers, as “rational economic people”, their subjective will and choice of land production mode are the main influencing factors of cultivated land resource utilization and management and grain production capacity. Moreover, Wang and Tian et al. found that the price of agricultural products directly affects the type of cultivated land utilization and the result of grain production [106]. At present, there is a realistic situation that is not optimistic; that is, the economic benefits of food production are generally lower than those of other economic crops. Therefore, when there is no government subsidy or it is too low, farmers’ willingness to plant food will continue to decrease and then switch to other economic crops [107]. In recent years, scholars have recognized that the change of land use types poses a greater threat to world food security than the small reduction of cultivated land area, and called on the Government to take effective measures to curb the drastic change of modes of man-made land use, which will help to stabilize the production area of cultivated food [108].

In addition, facing the problems of land fragmentation, higher agricultural production costs, lower agricultural productivity and lower grain output, most countries in the world have generally used effective measures, promoting moderately intensive land and large-scale production and management to transform and upgrade the agricultural system, to reduce agricultural production costs and to improve agricultural benefits [109]. Moreover, with the deepening of people’s understanding of environmental pollution and biodiversity [110], scholars’ attention to agricultural benefits has increased social and ecological benefits from a single economic benefit to emphasis more on the comprehensive benefits of agricultural production [111]. In this way, improving agricultural benefits not only protects the ecological environment but also stabilizes food production and ensures global food security.

#### 3.3.2. Analysis of Frontier Trending Topics

**Figure 7:**
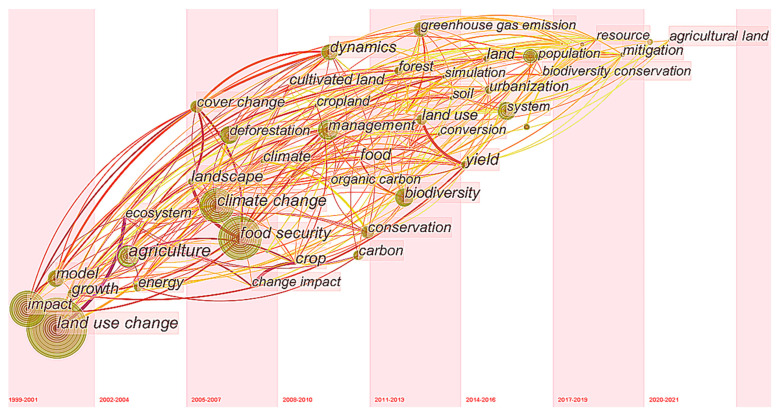
Time zone map for studying the evolution path produced by CiteSpace software.

Although the keyword clustering density map of the VOSviewer software can intuitively show the hot research topics in the field, the time factor is not considered. The time zone map of the CiteSpace software arranges keywords according to time series, which can more intuitively show the distribution of hot topics in each period [112]. Therefore, this paper combines the time zone map with the burst word detection function of CiteSpace software, which vividly shows the evolution of the research topic over time. We selected keywords with a frequency of more than five every one year (slice length = 1) from 1999 to 6 September 2021, to build a keyword cooccurrence network map (Figure 7). Furthermore, three burst words were detected (Table 6).

Combined with Figure 7 and Table 6, according to the time distribution of key nodes, we summarize the development trend in the research field of land use change and food security as follows: (1) The research in the field of land use change and food security started from the field of land use change and then combined the research with food security. (2) From 2009 to 2017, there were a large number of key nodes related to the theme of land use change and food security, including agriculture, land use, forest, rice, food demand and yield. During this period, research on land use change and food security was in a stage of rapid development, which once again shows that research on this topic has attracted the continuous attention of scholars. (3) In addition, scholars generally pay attention to key nodes, including climate change, carbon, urban expansion, and environmental impact, which suggests that scholars prefer to further analyze the impact of land use change on food security by researching the current situation and influencing factors of land use change. (4) Since 2017, scholars have paid more attention to global research on land use change and food security, raised the issue of food security to the field of risk research, and advocated the formulation and implementation of agricultural protection policies to ensure food security.

The burst detection algorithm was proposed by Kleinberg to explore the research frontier trend in a field by studying the strength and duration of keyword bursts [113]. The research on land use change and food security includes three top burst keywords: “area”, “consumption” and “ecosystem service” (Table 6). In 2015, the keywords “area” and “consumption” were the research hotspots, focusing on “differences in different research regions” and “energy and food consumption”, but the duration was short. In 2019, the keyword “ecosystem service” has become a new research hotspot and continues until now. It focuses on agricultural intensive development, grain production and environmental protection, which shows that ecosystem service will become a hotspot and trend in future research. As a whole, there are few burst words in this research field, indicating that the research concentration in this field is poor, and further research is needed.

## 4. Discussion

To ensure food security, countries all over the world have generally adopted strict land use restriction measures, such as China’s cultivated arable land minimum policy, Japan’s land classification management system and the United States’ land fallow policy. They hope to strictly restrict land use change through administrative control methods to stabilize food production and limit food risk within a controllable range [114,115,116]. At the same time, the academic research results on the theme of land use change and food security have increased significantly in the past two decades, and these results show a significant positive development trend.

### 4.1. Research Process

The results of this paper show that the first research article on the theme of land use change and food security was published in 1999. Since then, the number of published articles has shown a slow-growth trend. To deeply analyze the evolution of the research field of land use change and food security, this paper divides these research studies into three stages according to the number of articles published annually and the category and frequency of keywords. The first stage is the initial stage of research (1999–2008), during which the number of published articles was small, the research theme concentrated on a single topic, and overall research progress went slowly. Related research mainly focuses on conceptual and technical analysis, as well as direct analysis of the impact of land use change on food security. The second stage is the rapid development stage (2009–2017), during which basic research knowledge increased to a certain extent. The research focused on the influencing factors of land use change, including climate change, carbon emissions, and sustainable land management, climate change and sustainability would continue to be focused on in the future. During this stage, scholars also paid more attention to improving the ability to deal with land use change and food security risks, and to explore countermeasures and governance schemes at multiple levels, such as technology and policy. The number of articles published on the theme of land use change and food security did not increase significantly until 2009, which may attribute to the theme of World Food Day in that year, which was described as “coping with the crisis and achieving food security”, and emphasized the serious plight of malnutrition of 1.02 billion people in the world, as well as the need to help solve the problem of hungry people under conditions of economic crisis. The third stage is the stable deepening stage (2018–present). In this stage, as the global food security problem becomes increasingly serious, scholars pay more attention to global land use change and food security, and they raise the issue of food security to a risk problem.

### 4.2. The Impact of Land Use Changes on Food Security

We found that the research in this field mainly explores the impact on food security from four land use change factors: environmental change, land quality, crop planting type and agricultural production mode. First, environmental change involves climate change and carbon emissions. Excessive carbon emissions will lead to global warming and extreme climate events, resulting in changes in production factors such as moisture, heat, humidity, and temperature, which will lead to changes in land use patterns to varying degrees, eventually affecting the cultivated land area of food production and endangering food security. Second, land quality is related to land degradation. Due to the unreasonable use of land and changes in the natural environment, part of land in the world has experienced serious degradation (soil erosion), which leads to a decline in land productivity and has a serious impact on food production. Third, crop planting types involve renewable bioenergy and food production. Compared with the economic benefits of food crops, the economic benefits of other cash crops are higher, especially with the rapid development of clean energy (bioenergy), which leads to the conversion of some cultivated land originally planted with food crops to other cash crops. The reduction in the planting area of food crops will inevitably lead to a decline in total food production, then threatening global food security. Fourth, the agricultural production model involves sustainable land management policy, agricultural intensive development, food production and agricultural benefits. Facing practical problems such as land degradation, reduction of ecological land, land pollution and decline of soil fertility, people urgently need to change the extensive agricultural production model to the production model with higher overall efficiency. For example, the intensive agricultural model pays more attention to the stability of grain production and the protection of the ecological environment, which can give full play to the economic, social and ecological effects of agricultural production [117].

### 4.3. Research Hotspots

At present, the research topic in this field is mainly aimed at the complex practical problems of global land use change and food security, and the research content is becoming increasingly organized and systematic. According to the clustering results, we can extract seven frontier hot topics in this field: climate change and carbon emissions, sustainable land management policy, agricultural intensive development, land degradation, renewable bioenergy, food production and agricultural benefits. These results present the trend of cross integration with economics, land management, soil science, public policy, politics, geography, and other disciplines, and indicate that the research in this field continues to expand. Meanwhile, it is worth noting that although the theme of land use change and food security has become very popular in recent years, scholars’ research perspective is not limited to the direct analysis of the impact of land use change on food security but also considers climate change, carbon emissions, renewable bioenergy, agricultural intensive development models and other relevant aspects [7,8,54]. In terms of research methods, this field has formed a research system of geographic information technology, satellite remote sensing technology, theoretical models, investigations and interviews and other methods. Besides, through the analysis and summary of recent relevant literature, we found that scholars mainly focus on the hot issues including the utilization efficiency of chemical fertilizer (active nitrogen, etc.), urbanization expansion, greenhouse effects, and pay more attention to the combination of economic, social and ecological benefits of agricultural production to reduce the threat of land use change to food security [118,119,120,121].

### 4.4. Research Deficiency

This paper also has some research limitations that can be improved in the future. Firstly, because we only selected the WOS database as the data source for the bibliometric analysis of this study, and did not choose other databases (such as China National Knowledge Infrastructure, Scopus, et al.), the data used in our study cannot include all literature. However, as one of the most comprehensive databases in the world, the WOS database contains high-quality documents, it can represent the research hotspot and frontier in this field. Secondly, we can fully analyze the most influential publications in the database in the future, which will help to understand the real impact of the most important research in the scientific community.

## 5. Conclusions

This paper comprehensively used CiteSpace and VOSviewer software for bibliometric analysis and performed a visual analysis of the knowledge map of the literature with the theme of land use change and food security in the WOS database, and explored the research status, knowledge structure and evolution context. Since 1999, the number of annual published articles in the field of land use change and food security has shown an overall upward trend, which can be divided into three stages: initial research, rapid development, and a stable in-depth stage. Although a core author group was initially formed, the overall cooperation network is still relatively scattered. The distribution of research institutions is concentrated and forms a small cluster, which shows that there are only a few cooperative relationships among research institutions, and large institutional collaborative groups have not been formed across countries or regions. In national cooperation networks, developed countries are in the core position, but the cooperation network is not prominent. Meanwhile, keywords such as food security, land use change and climate change are taken as the core issues, they exhibit a radial shape and form seven frontier hot topics in this field. Moreover, due to the complexity of the research theme of land use change and food security, the research methods in this field are in-depth and diverse, and multidisciplinary development is constantly integrated. In addition, with the emergence of factors or problems such as climate change, carbon emission limitations, the application of new land use models and technologies, and the imbalance between food production and demand, there are new opportunities and challenges to the research on land use change and food security.

The research field of land use change and food security can be strengthened in the following aspects in the future. (1) Theoretical innovation still needs to be strengthened. At present, the research in this field is carried out through technology and mathematical models, which lack theoretical construction and innovation. The research perspective can also be innovated from the dimensions of land property rights systems and land management systems. (2) We should strengthen the research on the impact of agricultural chemical fertilizers on food security. Agricultural chemical fertilizer plays an important role in slowing down land use change and ensuring the sufficiency of food production, especially synthetic nitrogen fertilizer. However, the loss of active nitrogen will not only affect grain yields but also pollute the ecological environment. (3) Further research can focus on the impact of urban expansion on land use change. Due to the urbanization process of countries worldwide, a large amount of high-quality cultivated land around cities is occupied, which seriously threatens food security, especially in developing countries.

## Figures and Tables

**Table 1:** Author information table of the top 20 published articles.

Ranker	Count	Centrality	Year	Authors	Ranker	Count	Centrality	Year	Authors
1	16	0	2016	Peter H Verburg	11	5	0	2018	Wenbin Wu
2	13	0.03	2008	Pete Smith	12	5	0	2015	Isabelle Weindl
3	10	0.01	2014	Petr Havlik	13	5	0	2013	Alexander V Prishchepov
4	9	0.03	2014	Alexander Popp	14	5	0	2017	K Butterbachbahl
5	7	0	2014	Hermann Lotzecampen	15	5	0	2014	Hans Van Meijl
6	6	0	2014	Tomoko Hasegawa	16	5	0	2017	M C Rufino
7	6	0	2009	Jiyuan Liu	17	5	0	2017	Jasper Van Vliet
8	6	0	2014	Hugo Valin	18	4	0	2016	Almut Arneth
9	5	0	2014	Shinichiro Fujimori	19	4	0	2017	Kamini Yadav
10	5	0	2014	Christoph Schmitz	20	4	0	2014	Andrzej Tabeau

Note: The centrality indicator measures the importance of network nodes [44]. The larger the value of centrality, the more articles published by the author in cooperation with other authors.

**Table 2:** Information table of the top 20 major research institutions with published articles.

Ranker	Count	Centrality	Year	Research Institutions	Ranker	Count	Centrality	Year	Research Institutions
1	52	0.18	2003	Chinese Academy Science	11	12	0.1	2006	Potsdam Institute for Climate Impact Research
2	26	0.12	2014	Vrije University Amsterdam	12	12	0.01	2009	Beijing Normal University
3	22	0.22	2010	Wageningen University	13	12	0.03	2010	University of Edinburgh
4	19	0.02	2013	University of Chinese Academy of Sciences	14	12	0.03	2016	Karlsruhe Institute of Technology
5	18	0.13	2008	University of Aberdeen	15	11	0.05	2008	University of Maryland
6	18	0.12	2011	Humboldt University	16	10	0.03	2006	University of Copenhagen
7	16	0.1	2009	Michigan State University	17	10	0.02	2009	Lancaster University
8	14	0.09	2011	International Food Policy Research Institute	18	10	0.06	2000	Chinese Academy of Agricultural Sciences
9	13	0.07	2014	Commonwealth Scientific and Industrial Research Organization	19	9	0.04	2004	Columbia University
10	12	0.05	2000	International Institute for Applied Systems Analysis	20	9	0.03	2017	PBL Netherlands Environmental Assessment Agency

**Table 3:** Information table of the top 20 major research countries with published articles.

Ranker	Count	Centrality	Year	Countries	Ranker	Count	Centrality	Year	Countries
1	200	0.58	2003	USA	11	30	0.11	2010	France
2	125	0.1	2009	China	12	26	0.01	2007	Italy
3	114	0.13	2009	Germany	13	25	0.06	2013	Switzerland
4	93	0.11	2009	England	14	23	0.01	2011	Indonesia
5	92	0.1	2008	The Netherlands	15	22	0.02	2008	Belgium
6	56	0.04	2009	Australia	16	22	0.03	2008	Canada
7	47	0.04	2010	Scotland	17	20	0.09	2009	Denmark
8	36	0.02	2007	Austria	18	20	0.01	2009	Sweden
9	35	0.03	2008	Kenya	19	19	0.01	2015	Colombia
10	33	0	2009	Brazil	20	18	0.01	2012	India

**Table 4:** Information table of top 20 keywords with cooccurrence.

Ranker	Count	Centrality	Year	Keywords	Ranker	Count	Centrality	Year	Keywords
1	185	0.29	2000	Land use change	11	42	0.08	2009	Biodiversity
2	141	0.21	2006	Food security	12	41	0.07	2006	Deforestation
3	119	0.13	2006	Climate change	13	37	0.05	2010	Land use
4	102	0.08	1999	Impact	14	34	0.05	2000	Policy
5	62	0.14	2003	Agriculture	15	33	0.06	2008	Conservation
6	45	0.08	2009	Management	16	32	0.02	2014	Ecosystem service
7	45	0.04	2011	System	17	31	0.04	2012	Greenhouse gas emission
8	45	0.02	2000	Model	18	30	0.02	2006	Cover change
9	43	0.05	2006	Dynamics	19	30	0.08	2005	Carbon
10	43	0.06	2000	Pattern	20	27	0.02	2012	Expansion

**Table 5:** Keyword cooccurrence clustering induction.

Cluster-ID	Research Topics	Main Keywords Included
1	Climate change and carbon emissions	Climate change, global change, climate change mitigation, change impacts, greenhouse gas emissions, carbon sequestration, carbon stocks, greenhouse gas emissions, soil carbon sequestration, soil organic carbon
2	Sustainable land management policy	Land management, policy, protection policies, cropland protection, farmland abandonment, rapid urbanization, transformation, urban expansion, urban sprawl, urbanization
3	Agricultural intensive development	Agricultural intensification, sustainable intensification, agricultural productivity, ecosystem, environmental change, food security, biodiversity conservation, impacts, risk
4	Land degradation	Cropping systems, land use change, degradation, desertification, land degradation, pollution, soil erosion, water resources, croplands, climate change impacts, rice, river basin
5	Renewable bioenergy	Carbon, carbon footprint, water footprint, bioenergy, biofuel, energy, environmental impact, farming systems, life cycle assessment, production systems, renewable energy, soil erosion, sustainable agriculture
6	Food production	Crop productivity, crop yield, efficiency, food production, human appropriation, impact assessment, yield gap, use efficiency, net primary production, irrigation, maize, wheat
7	Agricultural benefits	Agriculture, benefits, biodiversity, certification, costs, crop, food demand, integrated assessment, intensification, plantations, policies, scenarios, validation, yields

**Table 6:** Top three burst keywords detection with the CiteSpace software.

Keywords	Year	Strength	Begin	End	1999–2021
Area	1999	3.56	2015	2016	▂▂▂▂▂▂▂▂▂▂▂▂▂▂▂▂▃▃▂▂▂▂▂
Consumption	1999	3.21	2015	2017	▂▂▂▂▂▂▂▂▂▂▂▂▂▂▂▂▃▃▃▂▂▂▂
Ecosystem service	1999	3.4	2019	2021	▂▂▂▂▂▂▂▂▂▂▂▂▂▂▂▂▂▂▂▂▃▃▃

## Data Availability

The data presented in this study are available in the Web of Science core database.
